# Atypical development of causal inference in autism inferred through a neurocomputational model

**DOI:** 10.3389/fncom.2023.1258590

**Published:** 2023-10-19

**Authors:** Melissa Monti, Sophie Molholm, Cristiano Cuppini

**Affiliations:** ^1^Department of Electrical, Electronic, and Information Engineering Guglielmo Marconi, University of Bologna, Bologna, Italy; ^2^Departments of Pediatrics and Neuroscience, Albert Einstein College of Medicine, Bronx, NY, United States

**Keywords:** causal inference, multisensory integration, ventriloquism effect, multisensory training, neural network, spatial sensory processing, autism spectrum disorder

## Abstract

In everyday life, the brain processes a multitude of stimuli from the surrounding environment, requiring the integration of information from different sensory modalities to form a coherent perception. This process, known as multisensory integration, enhances the brain’s response to redundant congruent sensory cues. However, it is equally important for the brain to segregate sensory inputs from distinct events, to interact with and correctly perceive the multisensory environment. This problem the brain must face, known as the causal inference problem, is strictly related to multisensory integration. It is widely recognized that the ability to integrate information from different senses emerges during the developmental period, as a function of our experience with multisensory stimuli. Consequently, multisensory integrative abilities are altered in individuals who have atypical experiences with cross-modal cues, such as those on the autistic spectrum. However, no research has been conducted on the developmental trajectories of causal inference and its relationship with experience thus far. Here, we used a neuro-computational model to simulate and investigate the development of causal inference in both typically developing children and those in the autistic spectrum. Our results indicate that higher exposure to cross-modal cues accelerates the acquisition of causal inference abilities, and a minimum level of experience with multisensory stimuli is required to develop fully mature behavior. We then simulated the altered developmental trajectory of causal inference in individuals with autism by assuming reduced multisensory experience during training. The results suggest that causal inference reaches complete maturity much later in these individuals compared to neurotypical individuals. Furthermore, we discuss the underlying neural mechanisms and network architecture involved in these processes, highlighting that the development of causal inference follows the evolution of the mechanisms subserving multisensory integration. Overall, this study provides a computational framework, unifying causal inference and multisensory integration, which allows us to suggest neural mechanisms and provide testable predictions about the development of such abilities in typically developed and autistic children.

## 1. Introduction

During everyday life, our nervous system has to deal with the multitude of stimuli coming from the surrounding external world. To produce a coherent perception of the environment, the brain needs to integrate information from different sensory modalities into a unitary perception, if produced by the same source. One of the main mechanisms that the brain exploits to achieve such a goal is multisensory integration (MSI), which denotes the brain’s capability to produce a better response to redundant congruent sensory cues, with respect to their single unisensory components.

Nevertheless, even if MSI is central to the nervous system’s ability to interact with such a complex external world, the integration of information from different sensory modalities is not always the best solution to interpret external events and it could lead to maladaptive behaviors when sensory signals originating from distinct sources and not causally related are integrated and not segregated. Therefore, the brain’s ability to correctly perform segregation and integration is as crucial as MSI for coherent perception of the multisensory environment we live in.

The problem of identifying when the stimuli come from a single source or multiple sources is usually referred to as the causal inference problem.

One of the most known and analyzed examples of this process is the speech-in-noise paradigm: when we are in a crowdy and noisy environment, to listen to and understand someone speaking the brain exploits the integration of auditory stimuli (the acoustic sounds correlated with spoken words) and congruent visual cues (lip movements) (Sumby and Pollack, 1954; Ross et al., 2007; Foxe et al., 2015). In this case, congruent auditory and visual information can improve the brain’s performance to detect spoken words and sentences. In the context of speech perception, another example of the brain dealing with the binding or segregation problem is the spatial ventriloquism illusion (Howard and Templeton, 1966; Bertelson and Radeau, 1981; Bertelson and Aschersleben, 1998; Alais and Burr, 2004; see Bruns, 2019 for a review). Moving an inanimate puppet’s mouth while speaking without moving his/her own mouth, a ventriloquist creates a powerful multisensory illusion leading us to perceive the voice as being produced by the puppet’s mouth. In this illusion, differently from the speech-in-noise condition, simultaneous spatially incongruent cues are incorrectly processed by the brain as belonging to the same event. The position of the visual stimulus alters the perceived location of a temporally congruent auditory cue due to the better spatial acuity of the visual system with respect to the auditory system, which instead is characterized by better temporal precision (Alais and Burr, 2004).

Even if the integrative abilities are fundamental to correctly navigating this multisensory world, optimal integration is not present at birth and appears to be dependent on post-natal experience. Several developmental studies characterized their maturational trajectories and how their acquisition is strictly related to the specific sensory experience gathered during infancy through adolescence. For example, Wallace et al. (2004a) found that kittens raised in the absence of visual cues were unable to combine cross-modal information. Moreover, animals exposed to altered sensory environments develop multisensory principles based on the unique sensory experience they are exposed to (Yu et al., 2010; Xu et al., 2014). These results highlight how the multisensory experience individuals are exposed to is critical to developing fully mature integrative capabilities. Specifically, a reduced multisensory exposure results in a delayed acquisition of MSI and integrative deficits (Yu et al., 2009; Xu et al., 2017; also consider Xu et al., 2014). Several studies highlight the existence of integrative deficits in children with a diagnosis of autism spectrum disorder (ASD) (see Baum et al., 2015 and Wallace et al., 2020 for recent reviews, but also O'Neill and Jones, 1997; Happé and Frith, 2006; Foss-Feig et al., 2010; Taylor et al., 2010; Cascio et al., 2012; Stevenson et al., 2012, 2014a,b,c; Brandwein et al., 2013; Wallace and Stevenson, 2014; Foxe et al., 2015, Beker et al., 2018). One possible explanation for reduced MSI in ASD is that these individuals experience a lower exposure to multisensory stimuli, possibly due to how attention is allocated (e.g., suppression of unattended signals; selectively focusing on one sensory modality at a time; not looking at faces consistently), as we tested and discussed in previous work (Cuppini et al., 2017b). Interestingly, while MSI deficits are found in children with ASD, they appear to be recovered in older subjects (Foxe et al., 2015; Beker et al., 2018). Indeed, adults with ASD show task performance matching those that neurotypical individuals (NT) reach already in late adolescence (see, e.g., Stevenson et al., 2018; Crosse et al., 2022).

Here we sought to investigate whether sensory experience contributes to the maturation of the ability to solve the causal inference problem, as this skill is strongly correlated with intact and mature MSI (Cuppini et al., 2017a,b). Although a wide array of research has focused on the integrity of MSI in Autism, the development of the capability of ASD individuals to solve the causal inference, and its relationship with multisensory processing, remains largely unexplored.

To the best of our knowledge, the presence of anomalous causal inference in the ASD population has been investigated only by one previous study (Noel et al., 2022), with mixed results, and in a limited age-related population (16.0 ± 0.5 years). Therefore, the underlying neural mechanisms and network architecture involved in these processes are not clearly identified yet.

To fill this gap, the purposes of the present computational study were (1) to extend the use of a previous neural model to study the developmental trajectories of causal inference, highlighting the underlying neural mechanisms, and how they can be affected by different sensory experiences; (2) to exploit this model to test the hypothesis that causal inference and its development are altered in ASD individuals, analyzing what mechanisms are involved. To address these questions, we exploited a neuro-computational model, based on physiologically plausible hypotheses, previously utilized to study multisensory interactions in ASD and causal inference (Cuppini et al., 2017a,b). We first assessed the relationship between multisensory experience and the development of causal inference. The developmental period was simulated by implementing a Hebbian learning algorithm and presenting the model with different percentages of auditory, visual, and congruent audiovisual stimuli. Results show the higher the exposure rate to cross-modal cues, the faster the acquisition of the ability to solve the causal inference problem. Next, we sought to investigate how this development is altered in the ASD population. ASDs’ causal inference development was simulated by assuming a lower multisensory experience at the beginning of the training, as suggested by previous computational work (Cuppini et al., 2017b). Indeed, altered MSI in autistic individuals has often been ascribed to their reduced attention, leading them to experience reduced multisensory exposure, which would compromise the emergence of integrative abilities, as discussed above. It has been hypothesized that at the beginning of puberty, thanks to several different reasons, among others changes in executive function and hormonal changes characterizing this phase of life, ASDs exhibit a renewed interest in the surrounding context and increase their social interactions. This, in turn, leads to increased experience with cross-modal events (Kuusikko et al., 2009; Beker et al., 2018). This is in accordance with cross-sectional studies suggesting a recovery of multisensory function as ASD individuals grow older (Foxe et al., 2015; Beker et al., 2018). To reproduce this scenario, we trained the ASD model by progressively increasing the percentage of audiovisual stimuli presented to the network. Similar to findings in multisensory paradigms involving speech stimuli or low-level cues (such as flashes and beeps), our results suggest that also causal inference reaches complete maturity much later in ASD individuals, compared to NT.

Overall, our study paves the way for future experiments aimed to test the model’s predictions and increases the knowledge of the autistic phenotype. Moreover, if verified experimentally, the results of our model suggest that children with ASD may benefit from multisensory training approaches, intended to favor a faster recovery of MSI and causal inference abilities.

## 2. Methods

**Figure 1:**
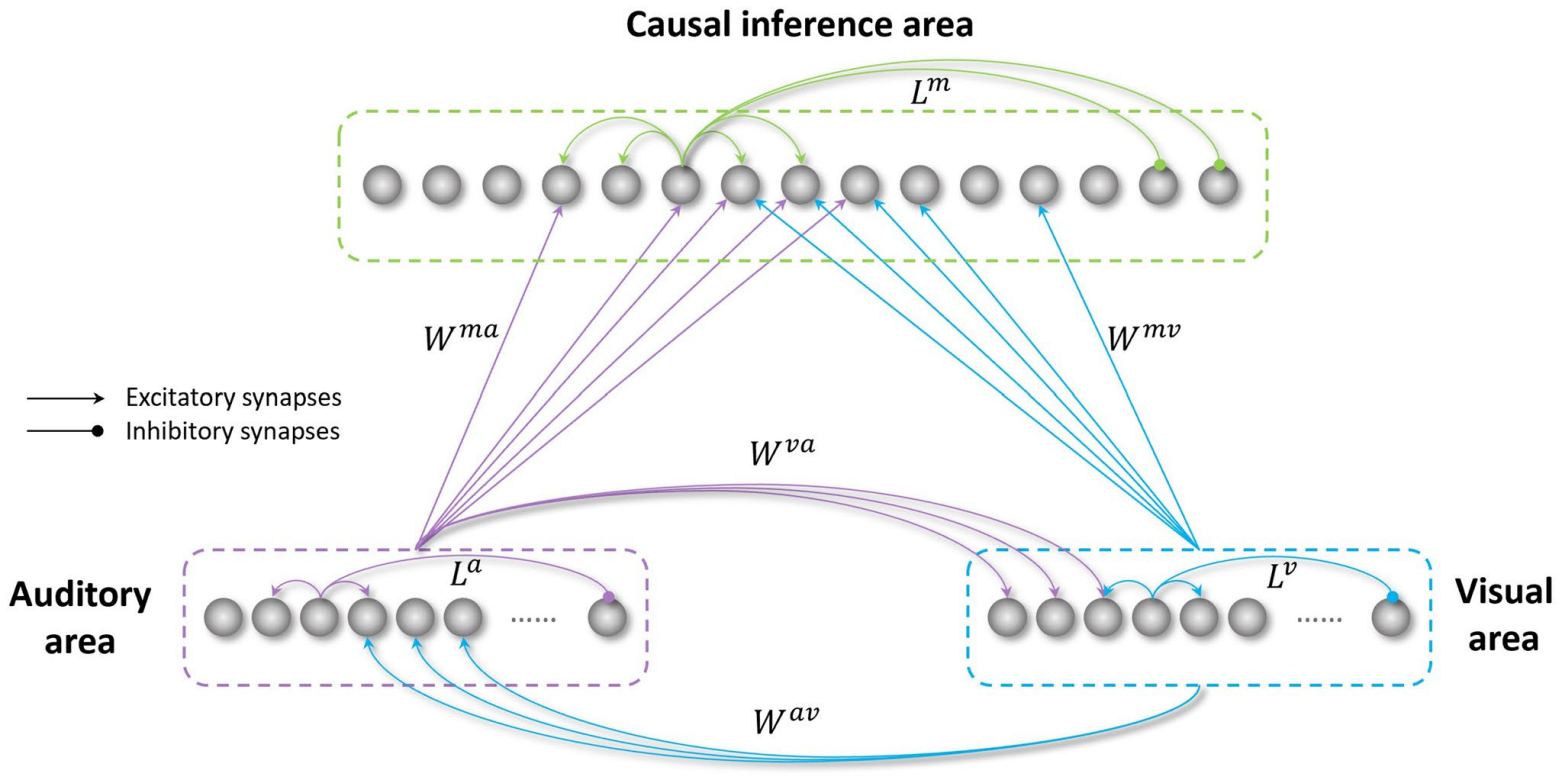
Structure of the network. The visual and auditory regions process external sensory stimuli. These regions are reciprocally connected through direct excitatory synapses ( $W^{av}$ and $W^{va}$ ), and send long-range feedforward projections ( $W^{mv}$ and $W^{ma}$ ), targeting the causal inference area. All these inter-area synapses are realized via Gaussian functions. The three regions in the network also include intra-area synapses (L), linking elements belonging to the same area. These connections are implemented using a Mexican hat function.

The model’s architecture (Figure 1) is based on a network previously realized to study the causal inference (Cuppini et al., 2017a) (the files and codes for this previous work are available online at figshare.com). The model was developed in Matlab.

In the following, the model structure, the implemented mechanisms, and the sensory training employed to mimic the transition from childhood to adult-like abilities are described qualitatively. Next, we provide the mathematical description of the model, including all equations, and the criteria for parameters assignment.

### 2.1. Model’s structure and mechanisms

The network consists of three areas: two unisensory regions (visual and auditory) processing the corresponding noisy external stimuli, and a multisensory region, responsible for the solution of the causal inference problem. Each area is made up of an array of *N* = 180 elements (the number of elements is arbitrary here), topographically organized; that is, proximal neurons code for proximal spatial positions. We assume a distance of 1° between adjacent elements. Neuronal responses to any input are described with a first-order differential equation which simulates the integrative properties of the cellular membrane, and a steady-state sigmoidal relationship simulating the presence of a lower threshold and an upper saturation for neural activation. The saturation value is set at 1, i.e., all outputs are normalized to the maximum. In the following, the term “activity” is used to denote neuron output. The topological organization in the regions is realized assuming that each element is connected with other elements of the same area via lateral connections (intra-layer connections, $L^{a}$ , $L^{v}$ , $L^{m}$ in Figure 1). These connections include both excitatory and inhibitory lateral synapses, which are arranged with a Mexican hat disposition (a central excitatory zone surrounded by an inhibitory annulus). Therefore, each neuron excites (and is excited by) its proximal neurons and inhibits (and is inhibited by) more distal neurons. Hence, activities of neurons belonging to the same region and stimulated by distal stimuli tend to suppress reciprocally (i.e., they interact via a competitive mechanism). For simplicity, in the network, we implemented the same lateral connectivity among elements in the unisensory regions, but we used a different connectivity in the multisensory layer to improve the solution of the causal inference.

Unisensory areas simulate the level of sensory processing performed in the unisensory cortical regions of the brain and are responsible for inferring the spatial location of the sensory stimuli. In the model, the perceived position of the external stimuli is obtained by computing the barycenter of the activities elicited in the visual and auditory areas, respectively. It is worth noting that we mimicked auditory and visual localization in the same way. This is a strong simplification, as the presence of a topographic organization is well documented in the primary visual areas, but it has not been observed in the auditory regions of the brain. This aspect is further discussed in previous papers (Cuppini et al., 2017a). Moreover, for this same reason, the acoustic area must be considered as functionally equivalent to several stages of processing in the auditory cortex. Elements in the unisensory regions also excite each other by means of reciprocal excitatory cross-modal synapses ( $W^{av}$ , $W^{va}$ ). These connections are symmetrical, and they are realized using a Gaussian function. Due to the presence of cross-modal projections, the inferred spatial localization of the auditory or visual inputs is affected by the concurrent presentation of the stimulus in the other sensory modality, even if the two events are processed separately in the two unisensory regions. From this point of view, the presence of the sigmoidal I/O relationship, with an “activation threshold”, is important. In fact, many neurons (especially in the auditory region) are silent but close to this threshold and can be easily excited by noise or cross-modal influences.

Accordingly, the net input reaching a neuron in the unisensory regions is the sum of three components: an external input, a multisensory input from neurons in the other modality (via cross-modal synapses $W^{av}$ , $W^{va}$ ), and a lateral input coming from other neurons in the same unisensory area (via lateral synapses $L^{a}$ and $L^{v}$ ). Moreover, to mimic the variability of sensory stimuli in a real environment, we added a noisy component targeting every element in the unisensory regions.

Information regarding the stimuli’ spatial configuration, extracted by these regions, is sent to a multisensory area (simulating an association cortex), for example, the anterior intraparietal sulcus, as shown by Rohe and Noppeney (2015a) through excitatory feedforward synapses ( $W^{mv}$ , $W^{ma}$ ), for which we used again a Gaussian function, identical for the two modalities. Units in the multisensory region also receive a lateral input, generated by the lateral synapses linking elements in the multisensory region ( $L^{m}$ ). The role of this region is to solve the causal inference problem. To answer this question, the activity elicited in the multisensory region is compared with a threshold (the “detection threshold”). The number of distinct peaks of activity, in the multisensory region, above this threshold identifies the number of distinct input sources inferred by the model. Stimuli placed in proximal positions (i.e., likely caused by the same event) excite proximal neurons in the multisensory region, producing a single peak of activity above the threshold. Conversely, stimuli from different spatial positions (i.e., likely generated by different events) stimulate distant multisensory neurons, eliciting multiple peaks above the threshold in the multisensory area.

Accordingly, the network presents two different levels of multisensory processing: the first, at the level of unisensory regions, mediated by the cross-modal synapses, influences the judgment about the spatial position of the external stimuli (some authors refer to this process as an “implicit causal inference,” see Odegaard et al., 2015, 2016; Rohe and Noppeney, 2015a; Odegaard and Shams, 2016; Noel et al., 2022; Shams and Beierholm, 2022); the second, performed in the multisensory region, is responsible for the solution of the causal inference problem (the “explicit causal inference” in Rohe and Noppeney (2015a), but see also Wallace et al., 2004b; Noel et al., 2022).

A further important mechanism in the model consists of competition/cooperation between elements in the same area. This is achieved via intra-area (lateral) synapses linking elements belonging to the same region, realized through a Mexican hat disposition, so that elements sensitive to proximal portions of the external world excite one another, and elements sensitive to different portions of the space are reciprocally inhibited. This synaptic arrangement concurs to identify the minimal distance between two activities in the same area that the network can separate, and thus are identified as produced by different events.

To summarize the role of the main mechanisms delineated above:

1. The two external inputs (auditory and visual) separately excite the two unisensory areas.
2. The cross-modal synapses between unisensory regions modify the spatial perception of the sensory inputs. In cases of proximal stimuli, which are usually perceived as originating from a common cause, the two positions are reciprocally attracted, thus generating typical perceptual illusions (such as ventriloquism). In cases of distant stimuli, which are usually perceived as coming from distinct input sources, these cross-modal synapses have a less important role, and intra-area inhibition becomes the dominant mechanism.
3. The feedforward synapses from the unisensory input regions realize a classic MSI, that is, the enhancement of the activities in spatial register. This is used to encode information on the mutual spatial coincidence of the cross-modal stimuli, and the likelihood that two stimuli were generated by a common source. Indeed, when two multisensory stimuli fall inside the RFs of the same multisensory neurons, the multisensory area integrates the information and presents a unique peak of activity, identifying a single input source.
4. The inhibitory lateral synapses implement a competitive mechanism, which allows the survival of the stronger stimuli only, while spurious or negligible stimuli are suppressed. This has two fundamental functions: it favors a spatial shift in the unisensory areas (where less reliable stimuli are shifted in the direction of the more reliable ones), and it engenders the effective elimination of unimportant sources in the multisensory area, where the readout of causal inference is effectively realized.

The visual and auditory inputs are described with a Gaussian function to mimic spatially localized external stimuli. The central point of the Gaussian function corresponds to the application point of the stimulus in the external world ( $\rho^{a}$ and $\rho^{v}$ , for the auditory and visual stimuli, respectively). The standard deviation of the Gaussian function ( $\sigma^{a}$ and $\sigma^{v}$ ) reflects the width of neurons’ RFs and the reliability of the external input, in other terms it mimics the spatial acuity of the two sensory modalities. Specifically, the two sensory modalities differ only for the width of the respective receptive fields and time constants. Indeed, because the visual spatial resolution is better than the acoustic one, visual receptive fields are smaller ( $\sigma^{v}<\sigma^{a}$ ). On the contrary, the better temporal resolution is the auditory one, therefore we chose ( $\tau^{v}>\tau^{a}$ ).

As for the temporal properties of the external stimuli, for simplicity in this work we chose synchronized auditory and visual stimuli, kept constant throughout the simulations (except for the analysis of the temporal window for the MSI, see below in Section 2).

### 2.2. Training the network

As stated in the Introduction, the first aim of this work was to test how the maturation of CI abilities depends on cross-modal experience. For this purpose, starting from the initial immature configuration, characterized by ineffective cross-modal projections (i.e., $W^{av}$ = $W^{va}$ = 0), we simulated the maturation of such connections, presenting the network with different percentages of unimodal and cross-modal inputs for a total amount of training epochs sufficient to reach an adult configuration.

In a first series of simulations, we run four different trainings, characterized by an increasing multisensory experience (Table 1).

**Table 1:** The training experience.

	AV (%)	A (%)	V (%)
Training 1	80	10	10
Training 2	60	20	20
Training 3	40	30	30
Training 4	20	40	40

Stimuli used during the training had a duration of 500 ms each and were generated through a uniform distribution of probability. We used stimuli at their highest level of efficacy, i.e., able to excite unisensory neurons close to saturation, in order to speed up the modeling process. During this period, both the intra-layer connections and the feedforward excitatory connections to the multisensory region were not subject to training. Therefore, we assume they are mature already in the initial configuration of the model. On the contrary, cross-modal synapses $W^{av}$ and $W^{va}$ were modified by using a simple rule for connection learning, consisting of Hebbian reinforcement and a decay term. In particular, the training algorithm reinforced the connections on the basis of the correlation between the activities in the pre-synaptic and post-synaptic neurons (Hebb rule). The decay term was proportional to the activity of the postsynaptic neuron and included a scaling factor that established the maximum saturation value for the connection.

It is worth noting that these values were not available in the literature, and presumably differ considerably across individuals and across the lifespan depending on circumstances. The first two trainings, performed with the highest multisensory experience, presumably resemble typical sensory experiences. Overall, each training lasted 8,000 training epochs, at which point the network showed steady-state configuration and performance.

The second goal of the work was to characterize the emergence of CI abilities in ASD subjects.

In a previous work (Cuppini et al., 2017b) we discussed that an altered/reduced exposure to cross-modal stimuli would be the most likely explanation for the delayed acquisition of integrative abilities in ASD children, compared to the two different hypotheses of a different level of synaptic plasticity, or reduced synaptic connectivity. Here, we assumed that a similar explanation could lead to altered maturation of the abilities to solve the causal inference problem in ASD. The sensory experience of ASD children was modeled by assuming a reduced exposure to multisensory stimuli. Moreover, ASD subjects naturally recover integrative deficit as they grow older (Foss-Feig et al., 2010; Taylor et al., 2010; Stevenson et al., 2012, 2014a,b,c; Brandwein et al., 2013; Wallace and Stevenson, 2014; Foxe et al., 2015; Cuppini et al., 2017b; Beker et al., 2018). Therefore, instead of performing the training of the simulated ASD subjects with a reduced and fixed percentage of AV stimuli, we chose to gradually increase the amount of AV stimuli presented to our network during the training phase. Accordingly, the fifth training performed to mimic the sensory experience of ASD subjects was characterized by the following sensory experience: 30% of AV stimuli during the first 2000 training epochs, 45% between 2000 and 4,000 epochs, and 60% from 4,000 to 8,000 epochs.

### 2.3. Mathematical description of the model

In the following, each neuron will be denoted with a superscript, *c*, referred to a specific cortical area (*c* = *a* or *v* or *m,* for the auditory, visual, or multisensory region, respectively), and a subscript, *j*, which indicates the spatial position within that area. *u*(*t*) and *y*(*t*) are used to represent the net input and output of a given neuron at time *t*. Thus, $y_{j}^{c}$ (*t*) represents the output of a unit at position *j*, belonging to the area *c*, described by the following differential equation:


(1) $$\tau^{c}\frac{dy_{j}^{c}\left(t\right)}{dt}=-y_{j}^{c}\left(t\right)+F\left(u_{j}^{c}\left(t\right)\right)$$

where $\tau^{c}$ is the time constant of neurons belonging to the area *c*, and $F\left(u\right)$ represents a sigmoidal relationship:


(2) $$F\left(u_{j}^{c}\right)=\frac{1}{1+e^{-s\left(u_{j}^{c}-\theta \right)}}$$

*s* and *θ* are parameters that establish the slope and the central position of the sigmoidal relationship, respectively. The saturation value is set at 1, i.e., all activities are normalized to the maximum. For the sake of simplicity, in this work neurons belonging to the three regions differ only for the time constants, chosen to mimic a quicker sensory processing for stimuli in the auditory region compared to visual stimuli.

The net input that reaches a neuron (i.e., the quantity $u_{j}^{c}\left(t\right)$ in Eq. 1) is the sum of two terms: a *within-region component* (say $l_{j}^{c}\left(t\right)$ ), due to the contribution of lateral synapses from other neurons in the same area, and a component coming from *extra-area sources* (say $i_{j}^{c}\left(t\right)$ ). Hence, we have:


(3) $$u_{j}^{c}\left(t\right)=l_{j}^{c}\left(t\right)+i_{j}^{c}\left(t\right)$$

To simulate the lateral input, $l_{j}^{c}\left(t\right)$ , neurons within each area interact via excitatory and inhibitory lateral synapses: each neuron excites (and is excited by) its proximal neurons, and inhibits (and is inhibited by) more distal neurons. Thus, the input $l_{j}^{c}\left(t\right)$ that a neuron receives from other elements of the same area is defined as:


(4) $$l_{j}^{c}\left(t\right)=\underset{k}{\sum }L_{jk}^{c}\cdot y_{k}^{c}\left(t\right)$$

where $L_{jk}^{c}$ is the strength of the lateral synapse from a presynaptic neuron at position *k* to a postsynaptic neuron at position *j*, both belonging to the same region *c,* and $y_{k}^{c}\left(t\right)$ is the activity of the presynaptic neuron at position *k* in the area *c*. These synapses are symmetrical and arranged according to a “Mexican hat” distribution (a central excitatory zone surrounded by an inhibitory annulus):


(5) $$L_{jk}^{c}=\{\begin{array}{c} L_{\mathrm{ex}0}^{c}\cdot e^{-\frac{{\left(d_{jk}\right)}^{2}}{2{\left(\sigma_{\mathrm{ex}}^{c}\right)}^{2}}}-L_{\mathrm{in}0}^{c}\cdot e^{-\frac{{\left(d_{jk}\right)}^{2}}{2{\left(\sigma_{\mathrm{in}}^{c}\right)}^{2}}}\;\mathrm{if}\;d_{jk}=0;\\ 0\;\mathrm{if}\;d_{jk}\neq 0\text{;} \end{array}$$

In this equation, $L_{\mathrm{ex}0}$ and $\sigma_{\mathrm{ex}}$ define the excitatory Gaussian function, while $L_{\mathrm{in}0}$ and $\sigma_{\mathrm{in}}$ the inhibitory one, $d_{jk}$ represents the distance between the pre-synaptic and post-synaptic neurons in the same area. To avoid undesired border effects, we use a circular structure to realize these synapses so that every neuron in each area receives the same number of side connections. This is obtained assuming the following expression for the distance:


(6) $$d_{jk}=\{\begin{array}{c} \left|j-k\right|\;\mathrm{if}\;\left|j-k\right|\leq N/2\\ N-\left|j-k\right|\;\mathrm{if}\;\left|j-k\right|>N/2\; \end{array}$$

In this work, we assume that both unisensory areas have the same pattern of lateral synapses, in order to limit the number of hypotheses in building the model. On the contrary, the lateral connectivity pattern in the multisensory layer is different, it was chosen to improve solution of the causal inference.

The external component of the input, $i_{j}^{c}\left(t\right)$ , has a different expression for the unisensory areas (*c* = *a*, *v*) and for the multisensory one (*c* = *m*).

The input to each *unisensory area* includes: a sensory stimulus from the external world (say $e_{j}^{c}\left(t\right)$ ), a cross-modal term coming from the other unisensory area (say $c_{j}^{c}\left(t\right)$ ), and a *noise component*, $n_{j}^{c}$ , realized by a standard uniform distribution on an interval $\left[-n_{\text{max}}+n_{\text{max}}\right]$ , where $n_{\text{max}}$ is equal to the 10% of the strength of the external stimulus for each sensory modality. Hence:


(7) $$i_{j}^{c}\left(t\right)=e_{j}^{c}\left(t\right)+c_{j}^{c}\left(t\right)+n_{j}^{c}\;c=a,v$$

The first term in Eq. 7 is simulated by means of a spatial Gaussian function, to reproduce the uncertainty in the detection of external stimuli. Assuming a stimulus of sensory modality *c* (*c* = *a* or *v*) presented in the position $p^{c}$ , the consequent input to the network can be written as:


(8) $$e_{j}^{c}\left(t\right)=E_{0}^{c}\cdot e^{-\frac{{\left(d_{j}^{c}\right)}^{2}}{2{\left(\sigma^{c}\right)}^{2}}}$$

where $E_{0}^{c}$ represents the strength of the stimulus, $d_{j}^{c}$ is the distance between the neuron at position *j* and the stimulus at position $p^{c}$ , and $\sigma^{c}$ defines the degree of uncertainty in sensory detection (which establishes the overall number of elements in region *c*, activated by the same external stimulus). As previously described for the lateral synapses, to avoid undesired border effects, also the external inputs are implemented as having a circular structure; hence, the distance $d_{j}^{c}$ is defined as:


(9) $$d_{j}^{c}=\{\begin{array}{c} \left|j-p^{c}\right|\;\mathrm{if}\;\left|j-p^{c}\right|\leq N/2\\ N-\left|j-p^{c}\right|\;\mathrm{if}\;\left|j-p^{c}\right|>N/2\; \end{array}$$

The cross-modal input, $c_{j}^{c}\left(t\right)$ , is obtained assuming that each unisensory neuron receives an excitation from the neurons processing the other modality. Hence:


(10) $$c_{j}^{a}\left(t\right)=\overset{N}{\underset{k=1}{\sum }}W_{jk}^{av}\cdot y_{k}^{v}\left(t\right)$$

$$c_{j}^{v}\left(t\right)=\overset{N}{\underset{k=1}{\sum }}W_{jk}^{va}\cdot y_{k}^{a}\left(t\right)$$

The weights of the excitatory cross-modal projections, $W_{ij}^{av}$ and $W_{ij}^{va}$ , were subject to the training. Thus, in the immature configuration, these connections were assumed ineffective.

The excitatory external input to the multisensory neurons is given by one contribution only, i.e., that due to the feedforward connections from the unisensory areas. Hence:


(11) $$i_{j}^{m}\left(t\right)=\overset{N}{\underset{k=1}{\sum }}W_{jk}^{ma}\cdot y_{k}^{a}\left(t\right)+\overset{N}{\underset{k=1}{\sum }}W_{jk}^{mv}\cdot y_{k}^{v}\left(t\right)$$

where $W_{jk}^{ma}$ and $W_{jk}^{mv}$ are the connections linking the presynaptic neuron at position *k* in the unisensory area (auditory and visual, respectively) to the neuron at position *j* in the multisensory area. These connections were not subject to the training phase, and are described by a Gaussian function:


(12) $$W_{jk}^{mc}=W_{0}^{mc}\cdot e^{-\frac{{\left(d_{jk}\right)}^{2}}{2{\left(\sigma^{mc}\right)}^{2}}}\;c=a,v$$

where $W_{0}^{mc}$ is the highest level of synaptic efficacy, $d_{jk}$ is the distance between the multisensory neuron at position *j* and the unisensory neuron at position *k*, and $\sigma^{mc}$ defines the width of the feedforward synapses. In the model for simplicity, we set the feedforward synapses identical for the two modalities ( $W_{0}^{ma}=W_{0}^{mv}$ e $\sigma^{ma}=\sigma^{mv}$ ).

During this phase, cross-modal connections (i.e., connections $W_{ij}^{av}$ and $W_{ij}^{va}$ ) are trained with a Hebbian rule: this training modifies the synaptic weight based on the correlation between the presynaptic and postsynaptic activity. The training rule is the following:


(13) $$\Delta W_{jk}^{c_{1}c_{2}}=\gamma^{c_{1}c_{2}}y_{j}^{c_{1}}\left(y_{k}^{c_{2}}-\frac{W_{jk}^{c_{1}c_{2}}}{W_{\text{max}}^{c_{1}c_{2}}}\right)\forall c_{1}c_{2}=av,va$$

which is an Hebb rule with learning rate $\gamma^{c_{1}c_{2}}$ and a decay term depending on the actual strength of the synapsis $W_{jk}^{c_{1}c_{2}}$ and the highest value fixed for the synaptic reinforcement $W_{\text{max}}^{c_{1}c_{2}}$ . In particular, according to Eq. 13 at steady state we have:


$$W_{\text{max}}^{c_{1}c_{2}}E\left\{y_{k}^{c_{2}}\right\}=E\left\{W_{jk}^{c_{1}c_{2}}\right\}\forall c_{1}c_{2}=av,va$$

*E*{} is the expected value. Since the activities of neurons are normalized between 0 and 1, the previous equation signifies that each connection cannot overcome the maximum value $\Delta W_{\text{max}}^{c_{1}c_{2}}$ (that occurs when the presynaptic activity is close to 1, in 100% of cases).

According to Eq. 13, the postsynaptic neuron must be active to have a change of synaptic efficacy. When this occurs, a postsynaptic neuron (in region $c_{1}$ , and position *j*), with a high activity $y_{j}^{c_{1}}$ , modifies its targeting connections $W_{jk}^{c_{1}c_{2}}$ , shaping them based on the actual activity $y_{k}^{c_{2}}$ of the presynaptic elements (in region $c_{2}$ , and position *k*). Conversely, silent postsynaptic neurons with poor output activity do not appreciably modify their connections, even when the presynaptic neural element is active. Moreover, the maximum value fixed for each pair of long-range excitatory connections is introduced to implement a saturation in the synaptic reinforcement. Thus, connections linking two neurons in the unisensory regions are modified as follows:


(14) $$W_{jk}^{c_{1}c_{2}}\leftarrow W_{jk}^{c_{1}c_{2}}+\gamma^{c_{1}c_{2}}y_{j}^{c_{1}}\left(y_{k}^{c_{2}}-\frac{W_{jk}^{c_{1}c_{2}}}{W_{\text{max}}^{c_{1}c_{2}}}\right)\forall c_{1}c_{2}=av,va$$

with $\gamma^{av}=\gamma^{va}$ . $W_{\text{max}}^{av}$ = $W_{\text{max}}^{va}$ is the maximum saturation value that synaptic weights cannot overcome.

### 2.4. Parameters’ assignment

The values of all model parameters (Table 2) were assigned based on findings reported in the literature, in accordance with the criteria summarized below.

**Table 2:** Parameters values.

**Neurons**	**Inputs**
N = 180 θ = 20 *s* = 0.3 τ = 1 ms $\tau^{v}$ = 15 ms $\tau^{a}$ = 3 ms	$E_{0}^{v}$ = 27; $\sigma^{v}$ = 4 $E_{0}^{a}$ = 28; $\sigma^{a}$ = 32 $\eta_{\text{max}}$ = 10%
**Lateral synapses, unisensory areas**	**Lateral synapses, multisensory area**
$L_{\mathrm{ex}0}$ = 5; $\sigma_{\mathrm{ex}}$ = 3 $L_{\mathrm{in}0}$ = 4; $\sigma_{\mathrm{in}}$ = 120	$L_{\mathrm{ex}0}$ = 3; $\sigma_{\mathrm{ex}}$ = 2 $L_{\mathrm{in}0}$ = 2.6; $\sigma_{\mathrm{in}}$ = 10
**Feedforward synapses**	**Training parameters**
$W_{0}^{ma}=W_{0}^{mv}=$ 18 $\sigma^{ma}=\sigma^{mv}$ = 0,5	$\gamma^{c}$ = 5 $\cdot 10^{-5}$ $W_{\text{max}}^{c}$ = 1,4

#### 2.4.1. External inputs

The strength of the external visual and auditory stimuli (parameters $E_{0}^{v}$ and $E_{0}^{a}$ ) is chosen so that the overall input elicits a response in the upper portion of the linear part of the sigmoidal static characteristic (i.e., a little below saturation). Additionally, physiological evidence (see, for example, Recanzone, 2009) show that the visual system presents better spatial resolution than the auditory one. This was mimicked by setting $\sigma^{a}>\sigma^{v}$ . In particular, the value of $\sigma^{v}$ was assigned to have a very acute visual perception (with a few degrees uncertainty). The value for $\sigma^{a}$ was set to have a large ratio $\sigma^{a}/\sigma^{v}$ , according to our previous computational studies (Magosso et al., 2012; Cuppini et al., 2014).

Finally, the noise level was fixed to 10% of the stimulus strength for each sensory modality, as this value allowed us to highlight the involved neural mechanisms.

#### 2.4.2. Parameters of individual neurons

The central abscissa, θ , was assigned to have negligible neural activity in basal conditions (i.e., when the input was zero). The slope of the sigmoidal relationship, *s*, was assigned to have a smooth transition from silence to saturation in response to external stimuli. The time constants were set so that the auditory processing is faster than the visual one, that is, $\tau^{a}<\tau^{v}$ , in accordance with experimental evidence of auditory cortical neurons presenting shorter latencies than neurons in the visual cortex (Maunsell and Gibson, 1992; Recanzone et al., 2000).

#### 2.4.3. Parameters of the lateral synapses

Parameters which establish the width and the strength of lateral synapses in every area (i.e., $L_{\mathrm{ex}0}$ , $L_{\mathrm{in}0}$ , $\sigma_{\mathrm{ex}}$ and $\sigma_{\mathrm{in}}$ ) were assigned to simultaneously satisfy multiple criteria: (1) inhibition must be strong enough to warrant competition between two stimuli in the same area; (2) the balance between excitation and inhibition must avoid instability, i.e., an uncontrolled excitation which propagated to the overall area; (3) the width of inhibitory synapses is large in the unisensory regions, to realize a stronger competition between two inputs of the same modality; (4) conversely, the width of inhibitory synapses in the multisensory region is smaller, in agreement with the possibility to have the coexistence of two peaks of activity in case of external causally unrelated stimuli, even at a few degrees distance.

#### 2.4.4. Parameters of the inter-area synapses

The strength and width of the feedforward synapses was chosen to provide an input to the multisensory neurons close to the central portion of the sigmoidal I/O relationship, when the network is stimulated by a strong unisensory external stimulus, but in the upper part close to saturation when stimulated by two concordant multisensory stimuli. This corresponds to the classic principle of multisensory integration (*enhancement*, *inverse effectiveness*). In particular, with this choice we have *super-additivity* in case of weak unisensory stimuli, and *sub-additivity* in case of stronger stimuli.

The strength of the direct cross-modal synapses between the unisensory regions was assigned high enough so that these connections can affect the responses of neurons of the opposite sensory modality when these elements are near or just above the activation threshold. Nevertheless, these synapses are maintained sufficiently low so that an external stimulus in one sensory modality does not induce a phantom activity in the other modality-specific area.

#### 2.4.5. Parameters of the training phase

The learning factor ( $\gamma^{c}$ ) and the sensory experience used to train the model were adjusted to achieve a good fit between the model and the data about the TD maturation as reported in Foxe et al. (2015). In particular, the value of $\gamma^{c}$ was not subsequently varied for the simulations of ASD development.

### 2.5. Performance evaluation

As stated above, we designed this model to help clarify the neural mechanisms responsible for the maturation of the ability to solve the causal inference problem in NT and ASD children.

To this aim, first, we compared the outcomes of the first four training configurations, to investigate the dependence of the ability to solve the causal inference on the multisensory experience. Subsequently, the last training configuration, characterized by an increasing proportion of AV stimuli (simulating ASD children), was compared with the outcomes obtained from the first two training configurations (those performed administering the highest proportion of AV stimuli, 60% and 80%, respectively).

To do this, we performed several simulations in multisensory conditions, with auditory–visual stimuli presented to the network, with different spatial distances (0°, 5°, 10°, 15°, and 20°), and we evaluated the network performance at different moments of the training (simulating the developmental period). In particular, we kept the visual stimulus fixed in a specific position of the space, and we shifted the position of the auditory stimulus. For each stimulus configuration, we run 100 trials.

To evaluate the ability to solve the causal inference and to compare the so obtained developmental trajectories in the two groups, the behavior of the network has been analyzed in terms of the following:

1. The “Report of Unity” (RoU), referring to how often the network identifies a common cause (*C* = 1, i.e., one peak above threshold in the multisensory area), or two different causes (*C* = 2, i.e., two peaks above threshold in the multisensory area) for the two stimuli, as described by Wallace et al. (2004b). This index has been plotted vs. the spatial disparity of the multisensory inputs, to identify the likelihood that the model integrates or segregates two multisensory stimuli at different distances.
2. The “Auditory Perception Bias”, referring to the bias in the perceived position of an auditory stimulus when presented along with a visual stimulus in a different portion of the space. This index has been computed as the spatial disparity between the real position of the external auditory stimulus and the position evaluated by the model (i.e., the barycenter of the evoked activity in the auditory area), divided by the distance between the real auditory and the real visual stimulus. First, it has been computed in general condition, that is, without taking into account the number of sources identified by the network. Then, the computation has been evaluated separately in the two cases of a common cause (*C* = 1), and different causes (*C* = 2), to investigate the relationship between the perceived auditory localization and the number of inferred causes. Because the visual position is only barely affected by sounds, due to the higher visual acuity, the visual perception bias has not been reported. Results are then compared with those reported in Wallace et al. (2004b), Odegaard et al. (2015), Hairston et al. (2003), Rohe and Noppeney (2015b).

## 3. Results

First, several simulations were performed to characterize the model’s behavior in the case of multisensory stimulations and to analyze the role of the different neural mechanisms involved. Next, additional simulations were run to analyze the maturation of the ability to solve the causal inference problem under different sensory experiences during the training period. Finally, a series of simulations were performed to validate the model’s architecture and the implemented mechanisms and formulate testable predictions.

### 3.1. Model’s behavior

**Figure 2:**
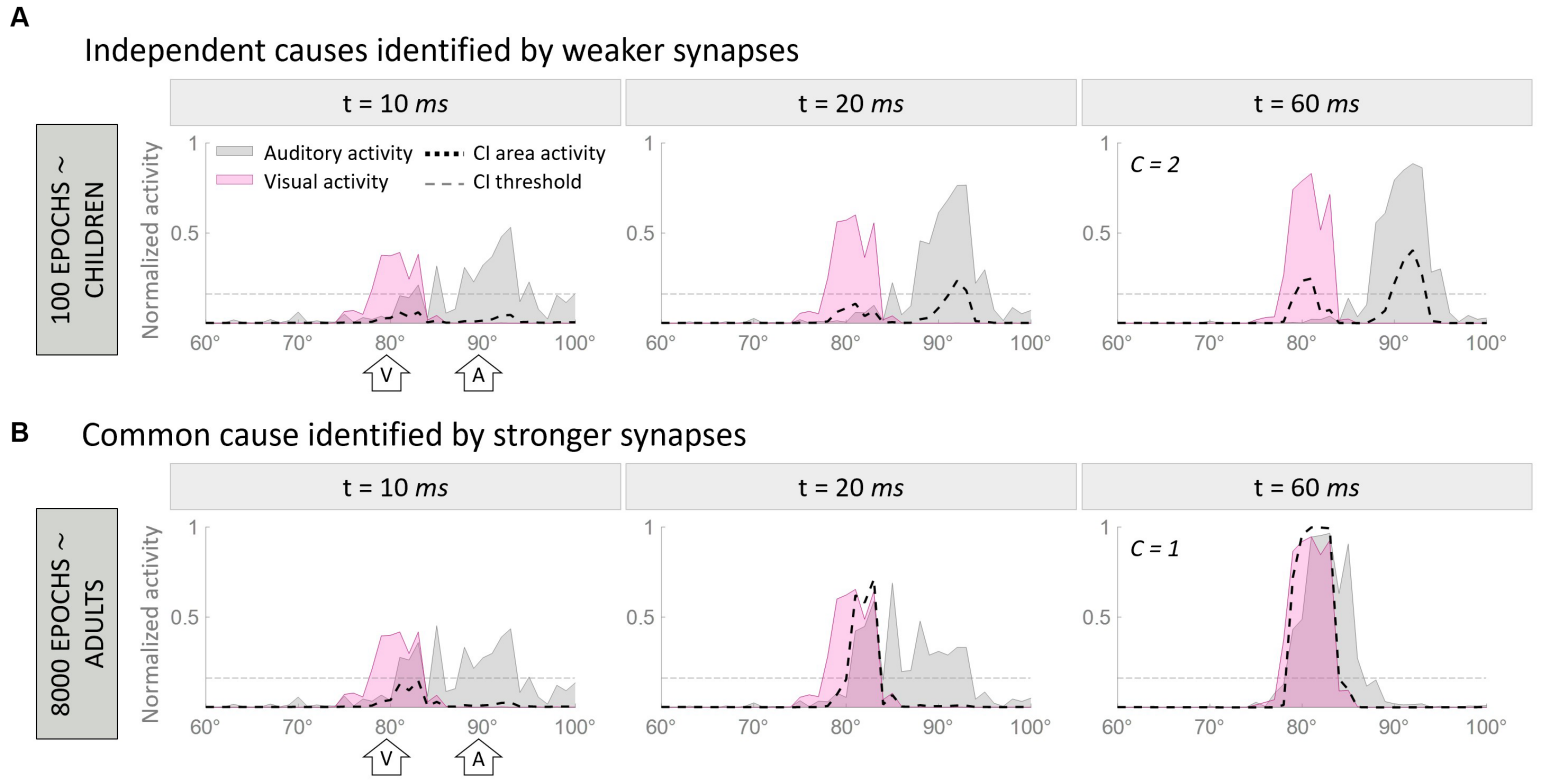
Temporal pattern of neural activity in the regions of the model. Panel **(A)** displays a simulation where two multisensory stimuli, separated by a spatial distance of 10°, are interpreted as originating from a single source. In contrast, Panel **(B)** presents an example where the same stimuli, with identical spatial configurations, are perceived as originating from separate sources due to the weaker cross-modal synapses. Within each figure, the gray area represents the activity in the auditory region, the pink is the activation of the visual region, and the black dashed line represents the activity of the multisensory area. Each panel consists of three columns, each representing a snapshot of network activity at different time points during the simulation. The left column reflects the initial stage of the simulation (10 ms), characterized by minimal activity below the threshold (gray horizontal dotted line in panels) in the multisensory area. The middle column corresponds to an intermediate moment (20 ms) when the threshold in the multisensory area has already been surpassed. Finally, the right column portrays the final configuration (60 ms) of the network activity.

To visually demonstrate how the model works, Figure 2 illustrates two representative cases. In particular, Figure 2 aims to clarify how the model handles multisensory cues, addresses the causal inference, and how its behavior evolves during training epochs. In this figure, we compare the model’s behavior after 100 training epochs, when the cross-modal synapses are still very weak, and at the end of the training, when the synaptic configuration is mature. This illustrates that the ability of the model to solve the causal inference problem changes over time, as a function of synaptic strength.

In both examples, the network is presented with an auditory stimulus at position 90° (corresponding to the 90th element of the region, the central one) and a visual stimulus 10° away from the auditory cue (i.e., at 80°). The input presented to the network is exactly the same in the two cases, therefore, the different results can be only explained by the different synaptic strengths of the cross-modal connections.

In the first simulation (Figure 2A), the two stimuli evoke activities in the corresponding unisensory regions centered on the neurons sensitive to the positions of presentation of the two inputs. These activities are minimally overlapping. The effect of immature cross-modal synapses is weak and unable to attract the two evoked activities and the correlated perceived positions of the stimuli. In this condition, auditory and visual activities elicit two distinct peaks in the multisensory layer (i.e., *C* = 2), and the model recognizes two independent sources, one for each sensory cue. Moreover, the distance between the perceived positions of the stimuli, computed as the barycenter of the evoked activities, is larger than the real one, as the auditory percept is repulsed by the visual one.

In the second simulation (Figure 2B), the two stimuli are presented in the same spatial configuration as the previous case, but during the simulation, the mature cross-modal synapses affect the activity evoked in the unisensory areas. More specifically, since the visual stimulus is more reliable in the case of localization tasks, the visual cross-modal component attracts the auditory evoked activity toward the position of the visual cue, altering the perceived position of the auditory stimulus, even if the external stimuli are presented 10° apart. In the end, the activities in the two unisensory regions largely overlap. With such overlapping evoked activities, both input regions send excitatory projections targeting the same neural elements in the multisensory area, producing a single peak of activity above the threshold.

Under these conditions, (1) our model identifies a single external event generating the two perceived stimuli (i.e., *C* = 1), and (2) the perceived position of the auditory stimulus is strongly attracted toward the actual position of the visual cue, thus resulting in a high auditory localization bias. In other words, the two stimuli are perceived as coming from portions of the external space close to each other and produced by the same event.

### 3.2. Developmental trajectories of TD children are a function of multisensory experience

**Figure 3:**
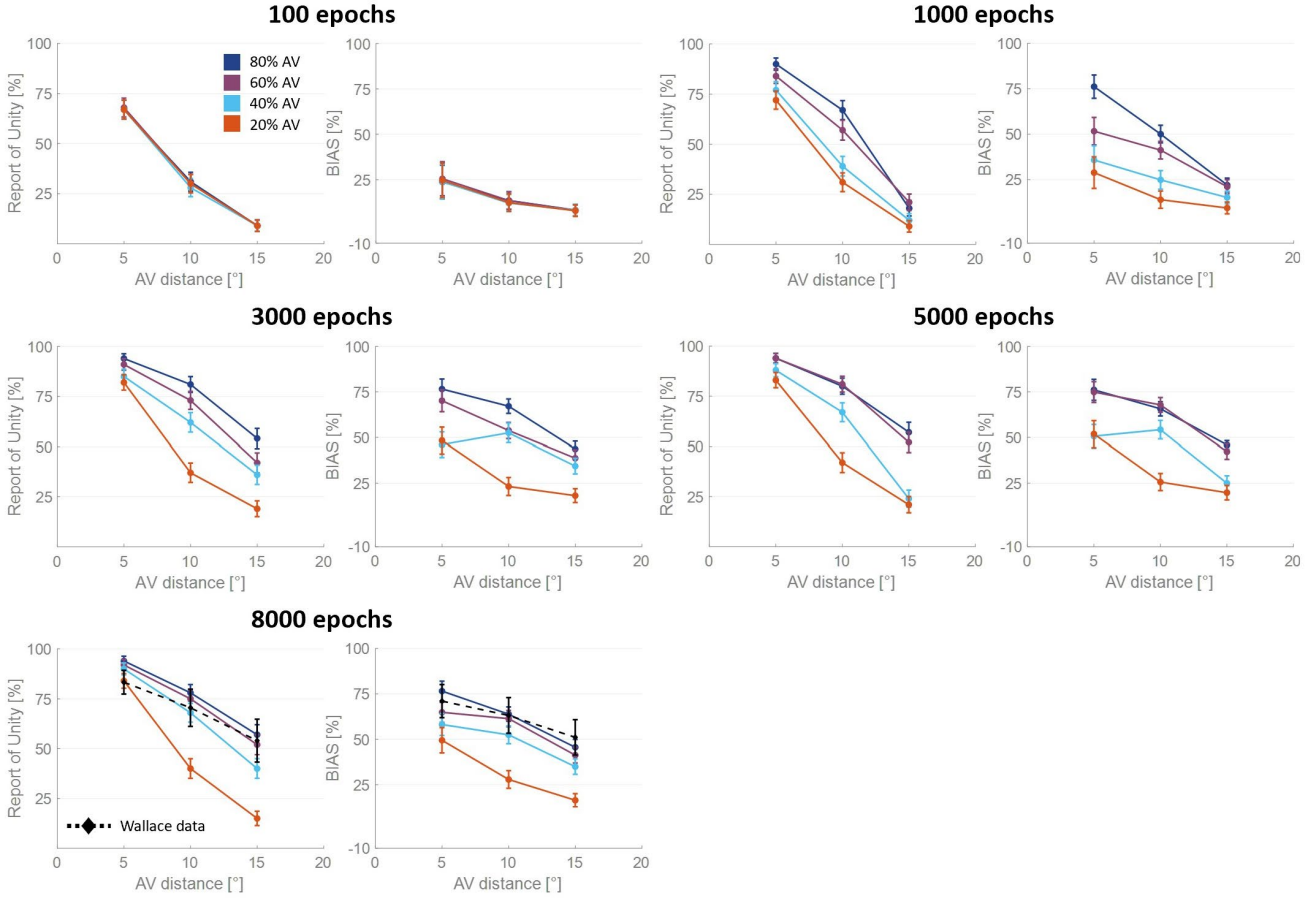
Percentual RoU and auditory bias as a function of AV spatial distance (in degrees) at different training stages. The input noise was set to 25% of the input strength and the detection threshold in the causal inference layer was 0.15. In each figure, different colors represent trainings involving different levels of multisensory experience. In particular, the blue curve has been obtained by training the model with 80% of AV stimuli, 60% of AV stimuli were presented to the network to obtain the purple curve, 40% for obtaining the light blue curve, and 20% for obtaining the red one. Both the RoU and the bias decrease with increasing spatial distance. Moreover, their values progressively increase during the training. It is worth noting that the RoU and the bias increase as a function of the amount of AV stimuli presented to the network. Particularly, the performance reached by the trainings conducted with over 50% of AV stimuli (blue and purple curves) is comparable. In contrast, the performance achieved by trainings involving a lower multisensory experience never catches up. Simulations’ results obtained at the end of the training (8,000 epochs) are compared with behavioral data from Wallace et al. (2004b) (black dashed line), obtained with an experimental paradigm similar to the one simulated by the model. The model’s results obtained by training the network with 80% or 60% of AV stimuli accurately fit these empirical data.

These observations prompted us to analyze more in detail how the auditory localization bias and the RoU change across training epochs (Figure 3). Both the report of spatial unity and the percent perceptual bias decrease significantly with increasing spatial distance. However, this general trend changes substantially over the training phase, as a function of multisensory experience.

For all four trainings, characterized by different levels of multisensory experience (20%, 40%, 60%, and 80%), the likelihood of reporting a common cause increases overtraining. After 100 training epochs, the model is still in an immature state, and no significant difference among the four trainings can be observed in the RoU. However, after 1,000 and 3,000 training epochs, multisensory experience and RoU are highly correlated. In particular, the increment of the RoU is more pronounced the higher the percentage of AV stimuli presented to the network. Between 3,000 and 5,000 training steps, the RoU attains its mature values for the training with 60 and 80% of cross-modal stimuli, and there is no further increase during the subsequent training. It is worth noting that the performance (as evaluated by the RoU) reached at the end of the training, is comparable when using over 50% of AV stimuli to train the model. Indeed, although the training performed with 60% of cross-modal stimuli progresses slower, the RoU values ultimately become comparable to those obtained when training the network with 80% of AV stimuli. In contrast, when the training is performed with 20% or 40% of AV stimuli (i.e., with a percentage of multisensory experience lower than 50%), the RoU continues to increase even after 8,000 epochs and never reaches levels comparable to those observed in the trainings with 60% or 80% of cross-modal cues.

Similar to the results just highlighted for the RoU, there is a consistent trend of increased auditory localization bias during the training, dependent on the level of multisensory experience utilized to train the network. Again, the cross-modal influence on the perceived position of the auditory cue is negligible after 100 epochs, for all four trainings. Yet, between 100 and 1,000 epochs a vision-capturing effect of hearing emerges clearly, and a relationship between perceptual bias and audiovisual exposure can be observed: the bias progressively grows, with a more pronounced escalation as the percentage of AV stimuli used to train the network increases. Between 3,000 and 5,000 epochs, the bias obtained with the training involving 80% of cross-modal stimuli reaches complete maturity. After 5,000 epochs of training, even the bias obtained from training conducted with 60% AV stimuli reaches its maximum values, comparable to the performance (as assessed by the bias) of the model trained with 80% multisensory stimuli. In other words, trainings with more than 50% of multisensory stimuli exhibit comparable bias values in the long term. The bias values achievable with the training involving 40% of multisensory stimuli, instead, partially recover between 3,000 and 8,000 epochs, reaching relatively high values, but still not comparable to those achieved with trainings using over 50% of cross-modal stimuli. Finally, training the model with 20% of multisensory cues leads to even lower performance.

### 3.3. Delayed developmental trajectories predicted for ASDs

**Figure 4:**
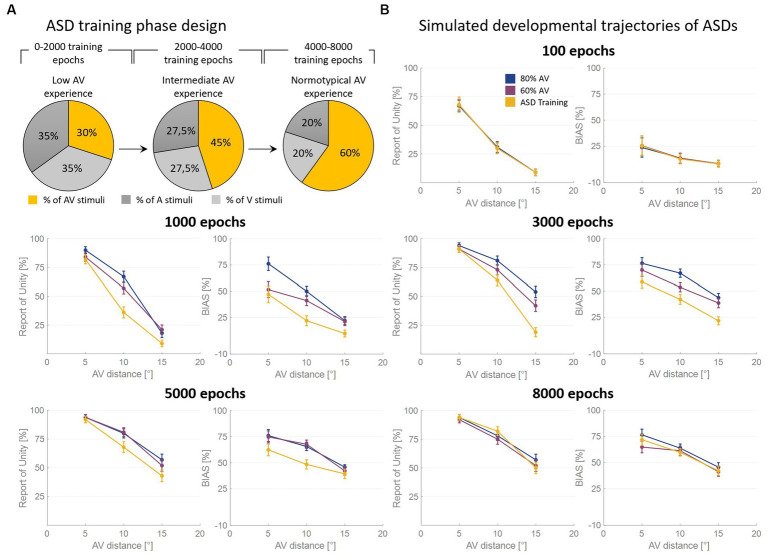
Simulated developmental trajectories of ASD children. Panel **(A)** illustrates the training process implemented for simulating ASDs development. Over the course of the training epochs, the proportion of AV stimuli increases to simulate the growing attention and exposure to multisensory stimuli experienced by ASD individuals during the developmental period. Panel **(B)** displays the percentual RoU and bias as a function of AV spatial distance (in degrees) at different training epochs. The input noise was set to 25% of the input strength and the detection threshold in the causal inference layer was set to 0.15. In each figure, different colors represent trainings involving different levels of multisensory experience. In particular, the blue and the purple curves represent trainings involving a fixed percentage of AV stimuli, equal to 80% and 60%, respectively. The yellow curve, instead, has been obtained by progressively increasing the proportion of AV stimuli presented to the network and is representative of ASD development. Both the RoU and the bias decrease with increasing spatial distance. Moreover, their values progressively increase during the training. It is worth noting that the RoU and the bias increase as a function of the amount of AV stimuli presented to the network: the blue curve is the first reaching full maturity, followed by the purple one, and, finally, by the yellow one. Notably, the final performance reached by the three trainings is comparable. This means that also the training performed by progressively increasing the multisensory experience is capable of achieving normotypical performance but with a delay of 2,000–3,000 training epochs.

As said, the RoU and the bias are comparable in the long term (from 5,000 training epochs on) when the model is trained with over 50% of AV stimuli. However, most notable is the finding that the training characterized by a progressive increase in multisensory experience (simulating ASD development, Figure 4A) achieves similar performances (Figure 4B). Nevertheless, both the bias and the RoU obtained through this type of training show a slower evolution compared to when the model is trained with a fixed and high proportion of AV stimuli. In fact, while trainings with 60% and 80% of multisensory cues reach full maturity between 3,000 and 5,000 training epochs, the RoU and bias resulting from progressive training only catch up to neurotypical performance at the end of the training period (8,000 epochs). In other words, progressive training is capable of achieving performance similar to that of training conducted with a fixed and high (>50%) proportion of AV stimuli, but with a delayed maturation of 2000–3,000 epochs.

### 3.4. Model’s validation

To the best of our knowledge, in the literature, there are neither studies dealing with the development of causal inference nor addressing this topic in ASD individuals. Therefore, we validated only the TD adult configuration of our model, while the developmental patterns of both TD and ASD represent valuable testable predictions to be tested in future behavioral experiments. We chose to compare the model’s results with the experimental conditions that are the most similar to our simulated configurations of stimuli. In particular, the experimental paradigm of Wallace et al. (2004b), who presented multisensory stimuli at varying spatial and temporal disparities, closely resembles the one simulated by our model. Also, the work by Odegaard et al. (2015) was conducted by presenting multisensory stimuli at different spatial locations, but the two unisensory components of the AV stimulus were always spatially congruent. Therefore, to compare our model with the results obtained by Odegaard and colleagues, we ran an additional set of simulations with an AV distance of 0°.

### 3.5. Simulations with increased AV spatial disparities

In their mature configuration, the two trainings with the highest proportions of cross-modal stimuli (60 and 80%) fit the pattern of empirical data of Wallace et al. (2004b). In particular, the bias decreases significantly with increasing cross-modal spatial disparity. However, a significant auditory bias (>45%) is still present at the largest simulated spatial distance (i.e., 15°). Similarly, also the RoU is influenced by the spatial relationship of multisensory stimuli. Indeed, also the RoU decreases substantially for increasing spatial disparities, even if unity is still reported on more than 55% of trials even at the largest spatial distance.

**Figure 5:**
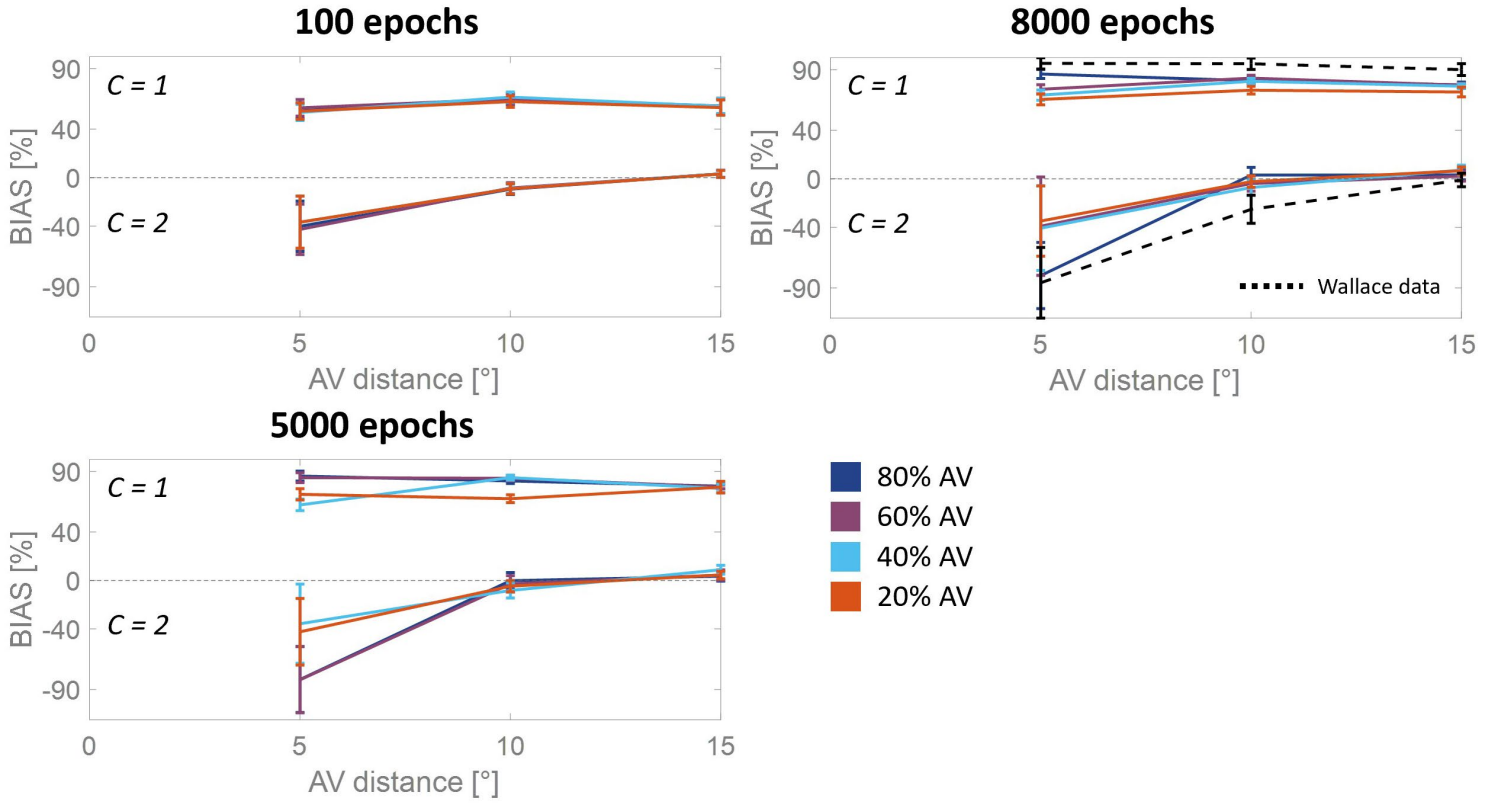
Auditory bias as a function of the AV spatial distance (in degrees) and number of sources, at different training epochs. The bias is examined separately when the network identifies a common cause (*C* = 1) or different causes (*C* = 2). The bias increases in absolute value during the training, as a function of multisensory experience: the higher the amount of AV stimuli presented to the network, the faster the development. At the end of the training process (8,000 epochs), the model trained with more than 50% of AV stimuli accurately reproduces the behavioral data from Wallace et al. (2004b) (black dashed line). In the case of *C* = 1, the bias is nearly complete and remains relatively consistent across various AV spatial disparities. However, when *C* = 2, the auditory bias is negative for distances smaller than 10°, while it is absent at larger disparities (i.e., 15°).

In accordance with what was stated by Wallace et al. (2004b), bias is a good predictor of RoU (i.e., whether perceptual unity will be reported or not). To further examine this relationship and how it evolves across epochs, we followed the same approach adopted by Wallace et al. (2004b) and we separated trials into two groups, those in which a single cause was identified and those in which two causes were identified (Figure 5). In the mature configuration (i.e., after 8,000 epochs) when the model recognizes a common cause for the two sensory cues, the bias attains very high values, signifying that the perceived position of the auditory stimulus is almost completely attracted toward the visual one, and the bias does not change consistently as a function of spatial disparity. In contrast, when the stimuli were not perceived as originating from the same source, the auditory bias strongly depends on the spatial disparity between stimuli. Specifically, no bias is observed at the largest spatial distances, whereas an increasingly negative bias emerges when stimuli are in close proximity to each other. This pattern of behavior is the same observed experimentally (Wallace et al., 2004b).

As is clearly evident from Figure 5, although the bias values at the beginning of the training are substantially smaller than in the mature configuration, the bias is already independent of the spatial disparity when spatial unity (*C* = 1) was reported. When the model does not report spatial unity (*C* = 2), the bias is strongly influenced by the distance between the stimuli, even at early development stages. Particularly, the bias is already negligible for the largest spatial distances, but it is significantly less negative than in adults when the sensory cues are close to each other. This pattern reflects the progressive development of the cross-modal synapses: as these connections become stronger, activities generated by close stimuli would progressively more likely be reciprocally attracted, producing overlapping activities in the two unisensory regions and eliciting the inference of a common source. This translates into a less negative bias in the case of independent sources inference for immature states than at the end of the training.

**Figure 6:**
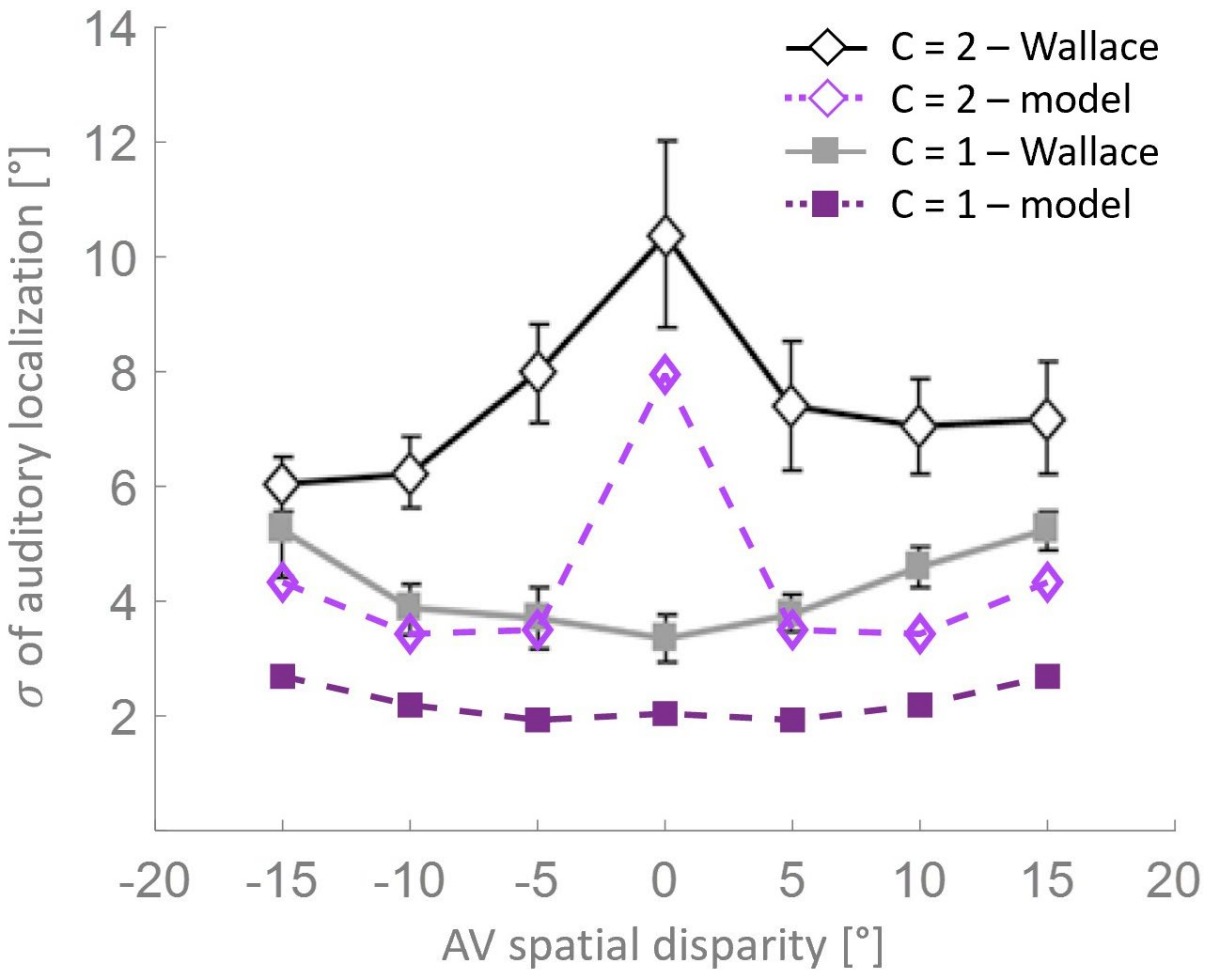
Localization variability as a function of spatial disparity. According to the model’s predictions (purple lines), when a single cause is identified (solid squares), the level of variation in stimulus localization, as measured by the standard deviation, is relatively low and increases only slightly with the spatial disparity between the stimuli. Conversely, when distinct causes are reported (open diamonds), the variance in stimulus localization is consistently and significantly higher compared to the single-cause case. This pattern of results resembles the behavioral data from Wallace et al. (2004b).

Wallace et al. (2004b) also found an interaction between the variability in localization and the RoU (Figure 6). Similarly, in the mature configuration of our model, when a single cause is not reported the localization variability is maximum for spatially coincident stimuli, and it is significantly lower for larger AV spatial disparities. When, instead, a common cause is identified, the localization variability is relatively small and increases with the spatial distance. Although the model can reproduce the general trend highlighted by Wallace et al. (2004b), the variability values obtained with the model are systematically lower than the experimental ones. This difference can be explained by the particular paradigm implemented by Wallace et al. (2004b), who investigated the causal inference problem both in the temporal and the spatial domain, presenting stimuli that are both spatially and temporally incongruent. Conversely, the stimuli we presented to the model are always simultaneous, even if spatially disparate.

### 3.6. Simulations with no spatial disparities

**Figure 7:**
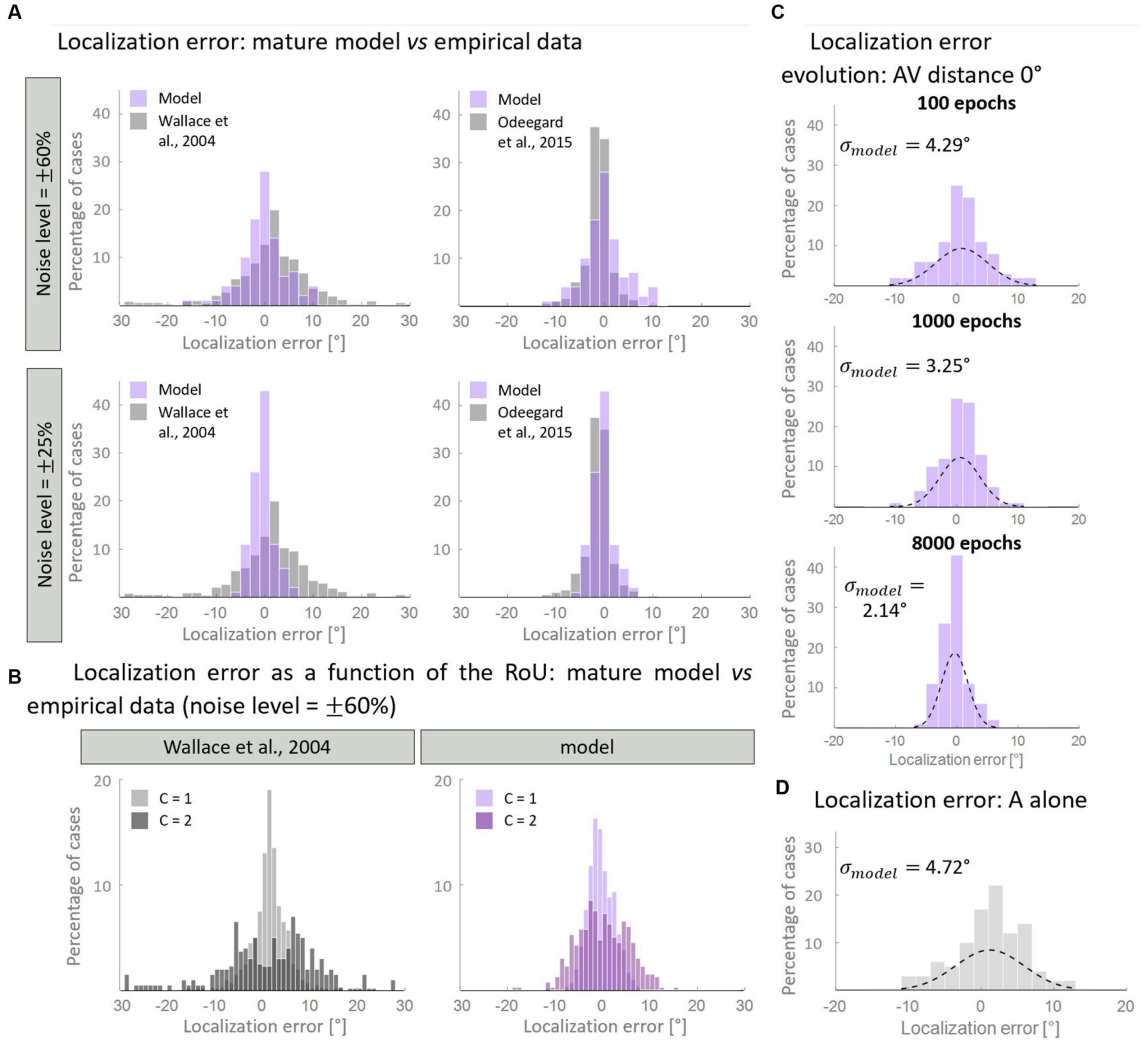
Distribution of the auditory localization error. Panel **(A)** compares the empirical distribution of localization error with that obtained with the mature model, trained with over 50% of AV stimuli. In particular, when the input noise is high, the model’s results are comparable with the data from Wallace et al. (2004b) (left column). A lower noise level is enough for reproducing the localization error distribution obtained by Odegaard et al. (2015) (right column). Panel **(B)** displays the same distribution, plotting separately the results for *C* = 1 and *C* = 2. Both our computational simulations and data by Wallace et al. (2004b) revealed a much broader distribution of localization error when two distinct causes are identified (*C* = 2), compared to when unity is reported (*C* = 1). Panel **(C)** shows the development of the distribution of localization error predicted by the model in the multisensory condition. Throughout the training epochs, the distribution of the localization error shrinks around 0°. Panel **(D)** reports the distribution of the localization error when only the auditory stimulus is presented to the network.

A more detailed analysis of the model’s results obtained with spatially coincident stimuli is reported in Figure 7. As previously stated, this stimuli configuration allowed us to test our model against additional experimental data, in particular those of Odegaard et al. (2015).

First, we aimed to determine if the mature configuration of the model accurately replicates the empirical distribution of localization error. Figure 7A illustrates the comparison between the model’s results and experimental data. Notably, by adjusting the level of input noise, the model demonstrates the ability to replicate distinct behavioral data. In particular, a higher level of noise is necessary to reproduce the localization distribution of Wallace and colleagues, whereas a lower noise level is sufficient to reproduce the data of Odegaard et al. (2015). A comparison with other empirical studies, in terms of standard deviations, is reported in Table 3. Next, we sought to test whether the model is capable of reproducing an additional finding of Wallace et al. (2004b). Particularly, these authors revealed that for spatially coincident AV stimuli, the pattern of localization responses is related to the RoU: when participants report a single cause, the localization error clusters closely around 0°; however, when two distinct causes are identified, the distribution of localization errors becomes broader. With an input noise equal to 60% of the stimulus strength (that is needed to reproduce Wallace data, as discussed above), our model is capable of reproducing this pattern of localization errors (Figure 7B).

**Table 3:** Variance of the auditory localization error.

Model (adult configuration)	Odegaard et al. (2015)	Hairston et al. (2003)	Rohe and Noppeney (2015b)
**AV (distance 0°)**			
σ= 2.14°	σ= 2.64°	σ= 1.5°	σ= 3°
**A alone**			
σ= 4.72°	σ= 3.01°	σ= 7°	σ= 3.68°

Model’s results are compared with empirical data. In all cases the standard deviation is reduced in the multisensory condition compared to the unisensory condition, although a large variability emerges across studies.

Finally, we investigated how the distribution of localization errors changes across epochs (Figure 7C). It appears evident that, in the multisensory condition, the distribution of the estimated A location becomes progressively more precise during the training, as it gradually clusters around 0°. This is particularly evident comparing the unisensory and multisensory distributions of the auditory localization error. While, at the beginning of the training, the standard deviation and the spatial distribution of auditory location estimate are comparable in the multisensory and unisensory conditions (Figure 7D), during the training we observe a significant improvement in the standard deviation of the auditory localization error (almost halved) in the multisensory case only. It is worth noting that the localization error in the unimodal auditory condition does not change during the training phase, as it is completely independent of the trained cross-modal synapses, and only depends on the dimension of the auditory receptive fields. Indeed, in a prior computational investigation (Ursino et al., 2017a), we provided evidence that the receptive fields of neural elements (both visual and auditory) undergo a reduction in size during training. This reduction enables the reproduction of spatial accuracy matching the respective sensory input and ultimately implements the likelihood estimate of unimodal spatial position. In this study, we made the simplifying assumption that the receptive fields are already fully developed at the start of the training process. As a result, considering that the receptive fields do not shrink over epochs, the unisensory localization abilities are also mature from the outset.

## 4. Discussion

The present work was designed with two fundamental goals: to study the effect of different sensory experiences on the development of causal inference and test the hypothesis that causal inference and its development are altered in ASD individuals due to an altered multisensory experience in childhood and adolescence.

In the literature, two approaches have been utilized to study the problem of how the brain deals with the problem of causal inference. One, namely the Bayesian approach, or Bayesian Causal Inference (Shams and Beierholm, 2022) assumes that the brain, first, infers the causal structure of the external stimuli, that is the number of sources/causes generating the signals (Alais and Burr, 2004; Shams et al., 2005; Körding et al., 2007). Then, based on the assumption of the most likely causal structure of the input sources and the perceived stimuli, it infers if the stimuli must be integrated or kept segregated (Wozny et al., 2010; Odegaard et al., 2015, 2016; Odegaard and Shams, 2016; De Winkel et al., 2018; Verhaar et al., 2022). In such a perspective, the tendency of the brain to integrate the different sensory information together to form a unitary percept is affected/based by the prior probability of common cause (p-common), that is the expectation of a common cause by the nervous system *a priori*, before receiving the sensory input (Alais and Burr, 2004; Shams et al., 2005; Körding et al., 2007; Shams and Beierholm, 2022). The second approach, namely the binding and segregation approach, suggests that the brain integrates different cues, or keeps them segregated, based on their features: for example, the “temporal correlation hypothesis” postulates that different aspects of the same event are bound together in the brain through the synchronization in the gamma range (30–100 Hz) of the activity of the corresponding neural representations. Synchronization of cortical oscillatory activity has been observed in response to visual, auditory and somatosensory stimuli and during different types of tasks (Damasio, 1989; Lebedev and Nelson, 1995; Singer and Gray, 1995; Singer, 1999; Varela et al., 2001; Brosch et al., 2002; Kaiser et al., 2002). To do that, the brain can utilize low-level features of the perceived sensory cues, such as the temporal and spatial properties, as well as high-level properties, such as semantic congruency. According to this approach, multisensory integration and cross-modal inhibition are mechanisms exploited by the brain to bind the perceived congruent sensory cues and to segregate the incongruent stimuli. In the first case, the brain would infer a common cause, in the second one it would recognize the stimuli as produced from independent sources. From this perspective, therefore, the solution to the causal inference problem relies on integrative abilities. Indeed, in a previous paper, we demonstrated that multisensory processing and sensory integration at the level of primary sensory cortices affects the ability to identify a single event or separate causes (Cuppini et al., 2017a). In different computational work, we also demonstrated that biologically plausible connectionist models can explain how the brain incorporates Bayesian concepts of the prior probability of common cause (p-common) and uncertainty about sensory cues (Ursino et al., 2017b) as the result of the developmental process.

These two previous works laid the groundwork for the development of a comprehensive framework able to describe and explain how sensory experiences affect the ability of the brain to solve the causal inference problem and form a coherent percept of the external world.

It is well known that the capabilities to integrate stimuli of different sensory modalities require experience with the environment, showing a slow maturational trajectory that can extend into late adolescence, and that is strongly affected by the specific sensory and multisensory experiences an individual has perceived. Even with such evidence about the reciprocal correlation between multisensory integration and causal inference and the maturation of the integrative abilities as a result of specific sensory experience (Yu et al., 2009; Xu et al., 2014), to our knowledge, no studies have been designed to analyze the acquisition of the ability to solve the causal inference problem in autistic subjects and in children in general.

To fill this gap in the literature, in this work we used a model, previously developed to analyze the ability of the adult brain to solve the causal inference, to make predictions about how sensory experience modulates the acquisition of such ability.

From our analysis two main predictions emerged. First, experience not only exerts a strong influence on the development of multisensory integrative abilities, but also affects the maturation of capabilities to identify the number of sources of the perceived external cues. More specifically, model simulations suggest that the brain needs a minimum multisensory experience (50% of audiovisual stimuli in our simulations) to reach a mature synaptic configuration able to support adult-like abilities to solve the causal inference problem. It is worth noting that with a low multisensory experience even a prolonged training period is not enough for the network to acquire fully mature behavior. Second, the acquisition of such abilities follows the maturation of the mechanisms underlying MSI. In our model, these mechanisms are identified by reciprocal cross-modal connections between primary unisensory cortices.

Given the lack of experimental evidence of how children perceive the causal structure of the environment, we validated our model and its predictions by comparing simulations’ results in the configuration at the end of the training with adult empirical data present in the literature. Despite its simple architecture, the model, in its adult configuration, effectively describes the relationship between spatial disparity and perceptual unification and reproduces the localization bias exerted by the visual modality on the auditory perception: both the bias and the report of perceptual unity increased for decreasing spatial disparity between the stimuli. Moreover, in a following set of simulations, we demonstrated that our model could reproduce several characteristics of MSI in the spatial domain. In particular, we examined the link between auditory bias and the report of unity. According to the literature, whenever individuals perceive an auditory and a visual stimulus as unified, the perceived position of the auditory stimulus is strongly attracted toward the actual localization of the visual one, independently of the real audiovisual spatial disparity (Bertelson and Radeau, 1981; Wallace et al., 2004b; Rohe and Noppeney, 2015a,b). Conversely, when the auditory and visual stimuli were judged as produced by different events, the auditory bias is negligible for spatially distant stimuli and becomes increasingly negative (i.e., the perceived location of the auditory stimulus is repulsed by the visual one) as the spatial disparity decreases (Wallace et al., 2004b). The mature architecture of the model proved to be capable of reproducing these experimental patterns of results. Then, we tested whether the model is able to reproduce the distribution of the localization error in the case of spatially coincident multisensory stimuli. It was sufficient to modify the noise level added to the sensory input, to reproduce different behavioral data from Wallace et al. (2004b) and Odegaard et al. (2015), reproducing also the dependence of the localization error on the number of perceived sensory sources: in case the model inferred a single source of the stimuli, the distribution of the perceived positions of the auditory components was centered around the real position of the stimuli (i.e., localization bias = 0°). Vice versa, in case of separate sources inferred by the model, the localization bias presented a more diffuse distribution, as shown also in empirical data (Wallace et al., 2004b).

It is worth noting that in recent years, numerous computational models have emerged with the aim of exploring how the brain solves the causal inference problem. Most of these computational models rely on the Bayesian approach and demonstrated that human behavior is nearly Bayes-optimal in a wide variety of tasks. Nevertheless, our understanding of the neural mechanisms underlying this optimality remains very limited. Our model suggests that Bayesian optimality might be implemented by our brain through the MSI mechanisms. By doing so, our model reconciles MSI mechanisms with Bayesian models and attempts to merge the two approaches into a unified framework.

As mentioned earlier, the second aim of this study was to investigate how altered multisensory experience of children with ASD could impact the development of their ability to solve the causal inference problem. In line with the study conducted by Cuppini et al. (2017b), we simulated the developmental pattern of ASDs by gradually increasing the proportion of audiovisual stimuli during the training period (as described in Section 2).

Our simulations suggest that an altered multisensory experience of children with ASD affects the maturation of their ability to solve the causal inference problem. The simulations also illustrate that these individuals reach adult-like performance later in life (at a developmental stage approximately coinciding with late adolescence or early adulthood), primarily due to the reduced exposure to multisensory stimuli, which prevents them from reaching the minimum multisensory experience, as discussed above, required for attaining adult-like abilities. It is important to note that at the beginning of the training designed to simulate individuals with ASD, the amount of multisensory experience provided to the network is lower than the minimum threshold. Indeed, in this initial training phase, both the RoU and the bias are lower for ASD individuals compared to their TD peers. However, as the training progresses, the percentage of audiovisual stimuli used to train the network gradually increases. Eventually, the multisensory experience reaches and overcomes the minimum threshold and drives improvements in performance. This indicates that the presence of a minimum threshold of multisensory experience, necessary for the development of fully mature integrative abilities, and the limited exposure to multisensory stimuli observed in individuals with ASD explain why their simulated developmental trajectories progress more slowly but ultimately reach the same level of performance as TD simulations.

The hypothesis of aberrant causal inference in Autism, and that it is linked to altered integrative abilities, is supported by abundant evidence for altered susceptibility of ASD subjects to multisensory illusions (other than the ventriloquism, see Table 4), in which the presentation of a stimulus in one sensory modality impacts the perception in another modality. Several studies (Taylor et al., 2010; Irwin et al., 2011; Bebko et al., 2014; Stevenson et al., 2014a) demonstrated that ASD children are less vulnerable to a well-known audiovisual speech illusion, the McGurk effect (McGurk and MacDonald, 1976; Saint-Amour et al., 2007). Contrary to the ventriloquism illusion, in the McGurk effect mismatching auditory and visual tokens of a speech syllable are presented such that they are spatially and temporally aligned as if they came from the same source. McGurk illusions refer to the influence that the incongruent visual stimulus has on the perceived auditory stimulus. Thus, an auditory “ba” presented with a video of someone articulating “ga” may be heard as “da” Stevenson et al. (2014b) found that ASD children are less vulnerable to non-speech audiovisual illusions as well, such as the sound-induced-flash illusion (SIFI) in which one visual flash is presented concurrently with multiple acoustic beeps, resulting in the illusion of perceiving multiple flashes. As such, the different susceptibility to audiovisual illusions is not due to difficulties of ASD subjects in processing socially relevant stimuli (such as speech) but is directly related to atypical multisensory processing. However, the literature provides conflicting results related to the susceptibility of ASD individuals to SIFI. Foss-Feig et al. (2010), for example, found a higher susceptibility to this illusion in ASD children, compared to their NT peers; whereas other studies (Van der Smagt et al., 2007; Keane et al., 2010) found no significant between-group differences. The different experimental paradigms used in these studies could explain the incoherent results. For example, the ratio between the number of flashes and the number of beeps used for producing the SIFI could have influenced perception. Indeed, Stevenson et al. (2014b) presented a single flash, coupled with several beeps, ranging between 2 and 4. On the contrary, the experimental paradigm used by Foss-Feig et al. (2010) allowed for a number of flashes that overcomes the number of beeps, thus establishing a second type of illusion, that is the fusion illusion (when multiple flashes are perceived as a single one when presented along with one beep). Moreover, different results could be motivated by different age groups as well: those studies that did not find any group difference used adult subjects (see Table 4 for further details); therefore, different behaviors could reflect different developmental stages of ASD subjects. Evidence that ASD children are less liable to be affected by cross-modal illusions is not restricted to auditory–visual interactions. In fact, ASDs exhibit a lower susceptibility to haptic-based illusions, such as the rubber hand illusion (RHI): the fake and the real arms must be stroked for a much longer time in ASDs relative to NT for this effect to appear (Cascio et al., 2012); moreover, the proprioceptive drift toward the rubber arm is significantly lower in ASD children (Paton et al., 2012).

**Table 4:** Main findings from studies on ASD populations involving multisensory illusions.

Study	# participants	Age (range, mean ± SD)	Paradigm	Main findings in ASDs
Children				
de Gelder et al. (1991)	17 ASD 17 TD	6.5–16.3 (10.9 ± 2.3) 6.8–11.1 (8.5 ± 1.3)	McGurk	Lower rate of the McGurk effect
Williams et al. (2004)	15 ASD 15 TD	5–13 (8.8 ± 1.4) 5–13 (9.5 ± 1)	McGurk	Less accurate in recognizing speech syllables in unimodal conditions, but normal integration of visual and auditory speech stimuli
Mongillo et al. (2008)	15 ASD 21 TD	8–19 (13.7 ± 3.9) 11–19 (13.4 ± 2.8)	McGurk	Impaired audiovisual MSI
Taylor et al. (2010)	24 ASD 30 TD	7.9–16.4 (12.4 ± 2.4) 8.3–16.4 (11.8 ± 2.5)	McGurk	Lower rate of the McGurk effect, but they “catch-up” with their NT peers as they grow older
Irwin et al. (2011)	13 ASD 13 TD	5–15 (9.1) 7–12 (9.2)	McGurk	Lower rate of the McGurk effect
Woynaroski et al. (2013)	18 ASD 18 TD	8–17 (12.3 ± 2.6) 8–17 (11.5 ± 1.9)	McGurk	Visual influence on heard speech over a wider time window (TBW)
Bebko et al. (2014)	15 ASD 19 TD	6.6–14.6 (10.5 ± 2.5) 6–15.6 (10.2 ± 2.7)	McGurk	Lower rate of the McGurk effect
Stevenson et al. (2014a)	32 ASD 32 TD	6–18 (11.8 ± 3.2) 6–18 (12.3 ± 2.3)	McGurk	Lower rate of the McGurk effect and wider TBW
Foss-Feig et al. (2010)	29 ASD 17 TD	8–17 (12.6 ± 2.6) 8–17 (12.1 ± 2.2)	SIFI (fusion and fission)	Higher susceptibility and wider TBW
Stevenson et al. (2014b)	31 ASD 31 TD	6–18 (12.1 ± 3.1) 6–18 (11.9 ± 2.9)	SIFI (fission)	Lower susceptibility
Cascio et al. (2012)	21 ASD 28 TD	8–17 (11.9 ± 2.8) 8–17 (13.4 ± 2.7)	RHI	Delayed susceptibility
Adults				
Keane et al. (2010) ^a^	10 ASD 9 TD	19–47 (30 ± 9) 18–49 (30 ± 8)	McGurk	No group differences
Van der Smagt et al. (2007)	15 ASD 15 TD	20.5 ± 3.2 20.7 ± 2.6	SIFI	No group differences
Keane et al. (2010) ^a^	10 ASD 9 TD	19–47 (30 ± 9) 18–49 (30 ± 8)	SIFI	No group differences
Bao et al. (2017)	20 ASD 20 TD	13–29 (18.7 ± 4.7) 13–28 (18 ± 9.5)	SIFI (fusion and fission)	No group differences for fission illusion, ASD more susceptible to the fusion illusion
Paton et al. (2012)	17 ASD 17 TD	32.06 ± 12.43 27.06 ± 6.2	RHI	Reduced susceptibility and lower proprioceptive drift toward the rubber arm
Palmer et al. (2013)	28 subjects, sorted based on their ASD-like traits	28.96 ± 11.16	RHI	The higher ASD-like traits, the lower the susceptibility
Mul et al. (2019)	22 ASD 29 TD	18–53 (27.1)	Full body illusion (FBI)	The higher ASD-like traits, the lower the susceptibility to FBI and self-location drift
Deltort et al. (2022)	15 ASD 15 TD	18–54 (28.5 ± 11) 18–54 (28.5 ± 10)	Enfacement illusion	Absence of enfacement illusion

For each of the two sections of the table (children and adults), studies on the dark gray background are those utilizing speech stimuli (McGurk effect), those on white background used lower-level sensory cues (SIFI), and those on light gray background focused on visuo-tactile illusions (RHI, full body illusion, enfacement illusion).

a Study that used both speech and non-speech stimuli and appears twice in the table.

Another argument in favor of the altered causal inference in Autism comes from the predictive strategy used by ASD individuals (Tarasi et al., 2022). Indeed, according to the predictive coding theory (Friston et al., 2014), perception is not merely driven by the incoming sensory inputs but is shaped by a prior knowledge, the prior internal mode. In the ASD population perceptual inference relies less on the prior, and it is rigidly shaped by the contextual sensory information gathered from the surrounding environment (sensory-driven behavior), thus overweighting the ascending prediction errors (Tarasi et al., 2022). In this framework, during the process of solving the causal inference problem, ASDs tend to underestimate the precision of their internal model, which is needed to link possible input sources to the perceived signals (i.e., to infer the causal structure of sensory events) and then impacts downstream processes. Therefore, ASDs are less likely to recognize a common source, which translates into a lower susceptibility to multisensory illusions.

Overall, these findings provide further support for the results obtained from our simulations, indicating that young children with ASD exhibit altered causal inference abilities. Additionally, most studies involving older individuals have failed to identify significant differences between the behavior of ASD and TD (Beker et al., 2018). This further strengthens our hypothesis that the capacity to infer the causal link between perceived sensory cues develops at a slower pace in individuals with ASD compared to TD, but eventually reaches a level of performance similar to TD individuals.

### 4.1. Limitations

The present study is designed as a purely computational work, as only the performance of the adult model (i.e., at the end of the training phase) was validated against experimental data. Therefore, the simulated developmental trajectories produced by our model do not represent definitive results, but rather testable predictions that suggest plausible neural mechanisms and guide future experiments. In the near future, experimental studies conducted directly on individuals with autism will be needed to confirm these results.

### 4.2. Future directions

Finally, we outline the future directions for investigations in this field. Currently, the model includes solely the mechanisms governing MSI in the spatial domain. However, in future works, we will enrich the model by incorporating the rules that govern the integration in the temporal domain. In a separate computational study (Cuppini et al., 2020) we already proposed a plausible synaptic architecture that accounts for MSI in the temporal domain. Therefore, an important advancement would be to merge the neural mechanisms of the present “spatial model” with the ones delineated by Cuppini et al. for the “temporal model.” This would result in a comprehensive computational framework capable of explaining and accounting for various tasks involving MSI in both time and space.

Bringing together the spatial and temporal domains is crucial, especially considering the well-documented temporal processing impairments observed in individuals with ASD. Specifically, individuals with ASD tend to have an expanded audiovisual temporal binding window. This means they perceive highly asynchronous stimuli as synchronous, attributing them to a single event (Brock et al., 2002; Foss-Feig et al., 2010; Stevenson et al., 2014b). Additionally, it is noteworthy that the temporal binding window undergoes developmental changes, even in typically developing individuals. It gradually decreases from childhood to adulthood, as observed by Hillock-Dunn and Wallace (2012), with adults having a smaller window compared to children and adolescents.

To conclude, we wish to point out that the model also suggests possible training strategies for the ASD population. Indeed, if the findings of this study are confirmed, it would be possible to explore rehabilitation strategies, specific for ASD individuals, based on multisensory stimulation, aimed at accelerating not only the development of multisensory abilities but also of causal inference abilities, which play crucial role in numerous everyday situations. Such a hypothesis is not purely speculative, and the result of this computational analysis is also confirmed by studies on animal models. For example, Stein and colleagues (Xu et al., 2017) showed that in multisensory neurons that did not develop integrative abilities, due to a lack of multisensory experience, a highly specific cross-modal training could speed up the acquisition of integrative abilities, if compared to an exposure to a normal environment, characterized by a higher sensory variability. Furthermore, it has been recently hypothesized (Stevenson et al., 2018) that perceptual and integrative deficits are at the origin of the core symptoms of autism (i.e., communication and social interaction deficits), therefore, it is possible that this rehabilitative strategy may also expedite the improvement of higher-level cognitive deficits, thereby significantly enhancing the lives of individuals with autism.

## Data availability statement

The original contributions presented in the study are included in the article/supplementary material, further inquiries can be directed to the corresponding author.

## Author contributions

MM: Conceptualization, Data curation, Formal analysis, Software, Writing – original draft. SM: Formal analysis, Supervision, Writing – review & editing. CC: Conceptualization, Formal analysis, Funding acquisition, Methodology, Software, Supervision, Writing – original draft.
